# Subpectoral Biceps Tenodesis Using an All-Suture Knotless Anchor

**DOI:** 10.1016/j.eats.2023.02.030

**Published:** 2023-05-15

**Authors:** Nathan Moroski, Joshua Eskew, Austin Cole

**Affiliations:** Prisma Health Blue Ridge Orthopedics–Seneca, Seneca, South Carolina, U.S.A.

## Abstract

The long head of the biceps tendon is a common pain generator in the anterior shoulder and is concomitantly seen with other shoulder pathology including subacromial impingement, as well as rotator cuff and labral tears. This Technical Note describes a mini-open onlay biceps tenodesis technique using all-suture knotless anchor fixation. This technique is easily reproducible, is efficient, and offers the unique benefits of providing a consistent length-tension relation and mitigating the risk of peri-implant reaction and fracture without sacrificing strength of fixation.

Long head of the biceps tendinopathy is a well-known generator of pain in the anterior shoulder.^1^^,^^2^ Multiple different causes of anterior shoulder pain stemming from biceps pathology exist. Frequently, biceps tendon pathology is seen concomitantly with other shoulder pathology including labral tears, rotator cuff disease, and impingement syndrome.^1^^,^^2^ Biceps tenotomy and tenodesis are common surgical treatment options when conservative measures fail. Multiple recent studies have shown decreased biceps function and cosmetic effects with tenotomy versus tenodesis.^3^^,^^4^ Tenodesis is the choice preferred by many surgeons, particularly for patients who are young and active. Many different techniques have been described varying in tenodesis location, fixation device, and use of an open versus arthroscopic approach. However, there is no clear consensus regarding the optimal treatment strategy.

Previous studies have examined both benefits and limitations regarding the spectrum of biceps tenodesis methods. Subpectoral tenodesis releases the tendon from the bicipital groove, eliminating potential sources of pain that originate from the proximal portion of the tendon or tenosynovium within the groove.^5^^,^^6^ Furthermore, reoperation rates have been shown to be lower with subpectoral versus proximal tenodesis.^7^, ^8^, ^9^ There has been no substantial evidence to date showing significant differences in clinical outcomes regarding fixation methods.^10^, ^11^, ^12^, ^13^ Nevertheless, interference screw fixation is known to be associated with an increased risk of humeral fracture, persistent pain, and patient reactions to screws.^5^^,^^11^^,^^14^, ^15^, ^16^ To mitigate these risks, some surgeons have transitioned to onlay techniques. Various studies have shown no significant difference in strength or failure, with good patient-reported outcomes.^11^^,^^13^^,^^17^ Recently, knotless suture anchor fixation has garnered increased attention in arthroscopy. It is important to note that comparable load to failure has been found using knotless suture anchors in relation to other devices.^12^^,^^18^

The purpose of this Technical Note is to describe a technique using knotless all-suture anchor fixation for subpectoral biceps tenodesis. We describe a mini-open onlay tenodesis that is simple and reproducible while avoiding the aforementioned complications of alternative fixation locations and devices.

## Surgical Technique

**Fig 1:**
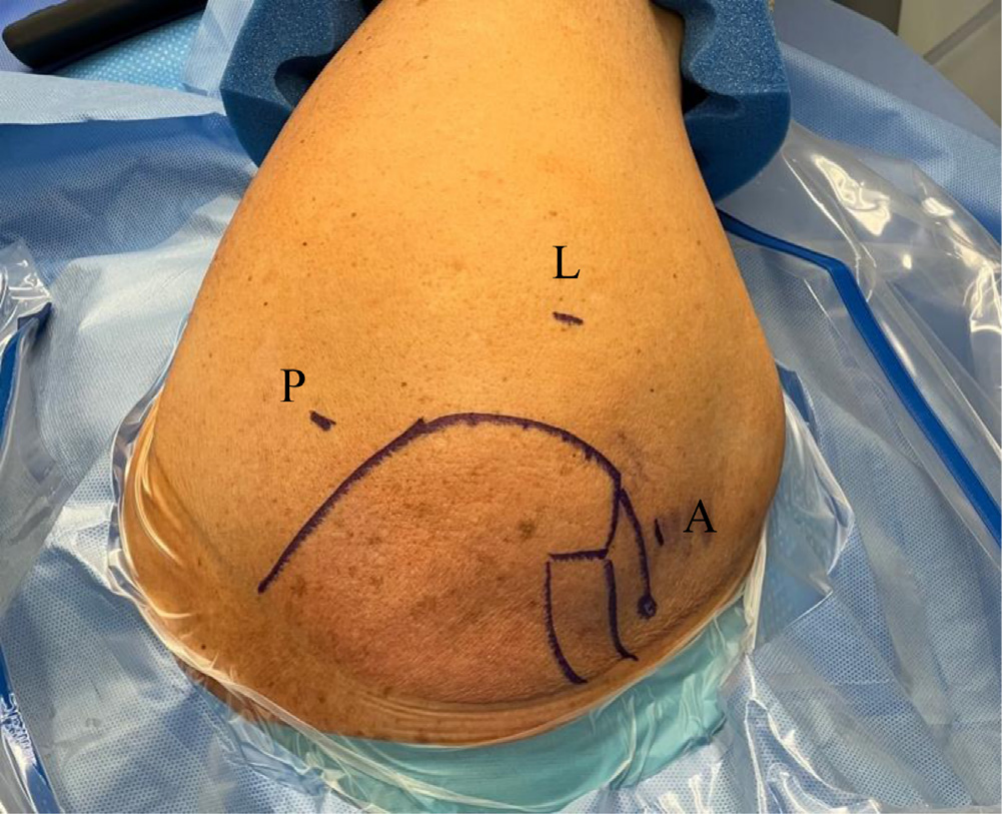
In a left shoulder, the patient is placed in the lateral decubitus position, and anatomic landmarks are delineated. The standard posterior portal (P) and direct anterior portal (A) are used for diagnostic arthroscopy and biceps tenotomy for later tenodesis. The lateral portal (L) is marked and can be used if necessary.

The patient is placed in the lateral decubitus position with the operative shoulder prepared and draped in the usual standard fashion. Two portal sites are used for the procedure: a standard posterior portal, 2 cm inferior and 1 cm medial to the posterior corner of the acromion, and a direct anterior portal (Fig 1).

After joint insufflation with 30 mL of saline solution using an 18-gauge spinal needle, the posterior portal is created using a No. 11 blade scalpel. The glenohumeral joint is then entered with a blunt trocar and sheath, and the scope is inserted. The anterior portal is created under direct visualization using a spinal needle, followed by the use of a No. 11 blade scalpel to incise the skin. A blunt trocar is used through the anterior portal to enter the joint. A probe is then placed through the anterior portal to aid in diagnostic arthroscopy. The glenohumeral joint and anterior, inferior, posterior, and superior labra are all carefully examined and probed. The rotator cuff is then examined for any pathology. Our attention now turns to the biceps tendon, which can be pulled into the joint using the probe to examine for tenosynovitis within the bicipital groove. After the decision for biceps tenodesis is made, arthroscopic scissors or a meniscal biter is placed through the anterior portal and used to perform a biceps tenotomy for later tenodesis. Any remaining biceps stump is debrided with a 4.0-mm shaver (Arthrex, Naples, FL). Any additional pathology in the glenohumeral joint and subacromial space is then addressed, and the arthroscope is removed from the shoulder.

**Fig 2:**
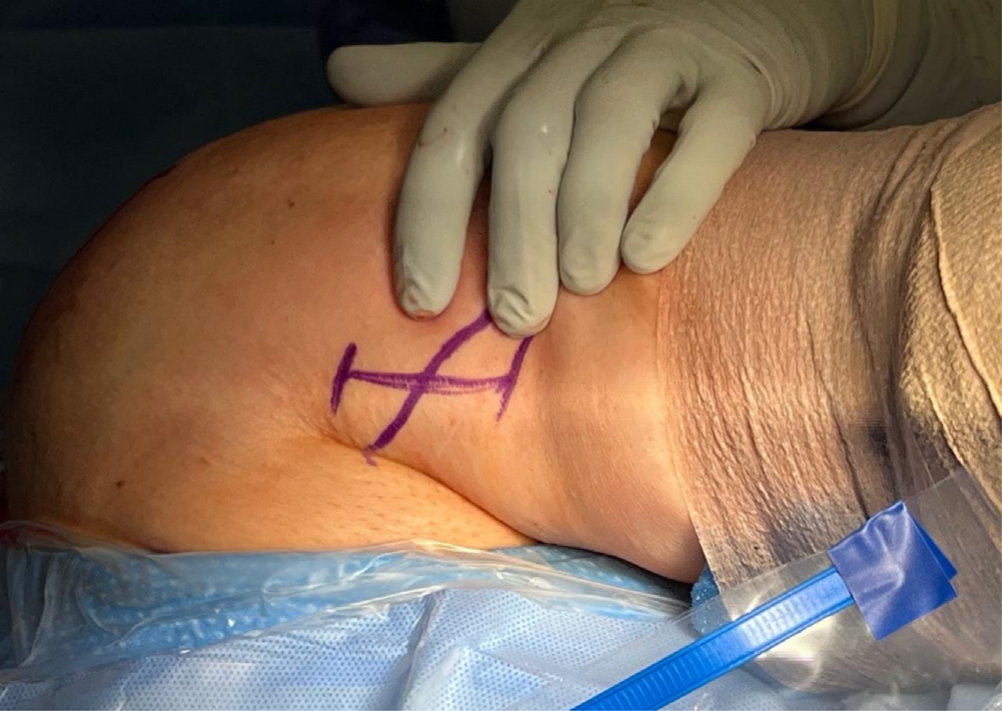
In a left shoulder in the lateral decubitus position, a biceps tenotomy is performed during arthroscopy. The patient remains in the lateral decubitus position, and the arm is extended 20°. A longitudinal incision is centered over the inferior border of the pectoralis major.

**Fig 3:**
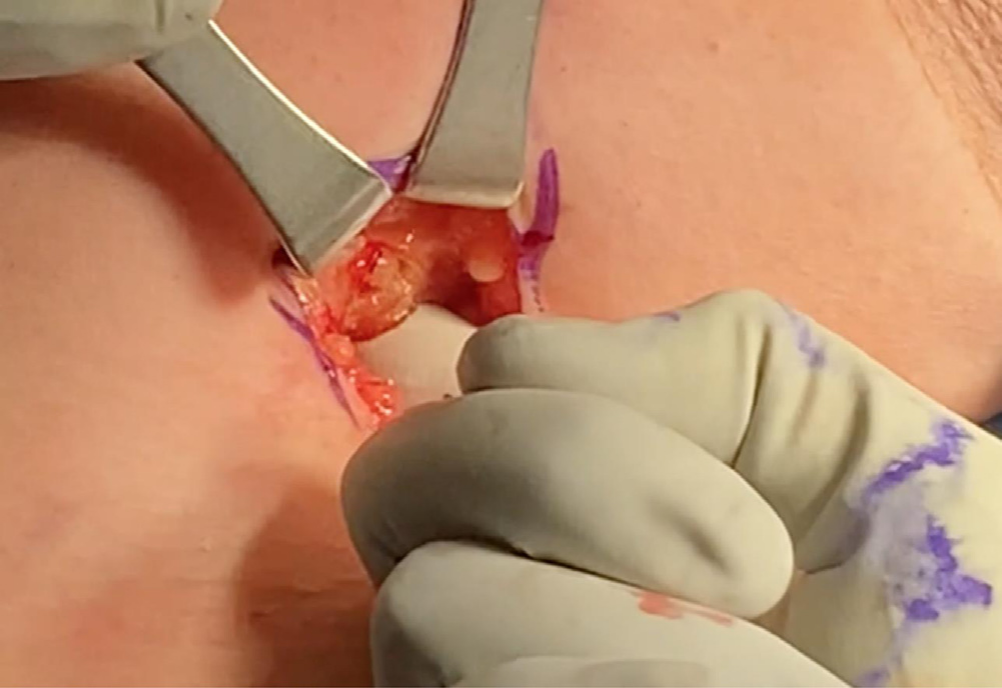
In the left shoulder in the lateral decubitus position, dissection is carried down through the subcutaneous fat in the shoulder. The fascia in line with the inferior border of the pectoralis major is then incised. Finger dissection is used to palpate the long head of the biceps tendon within its groove.

**Fig 4:**
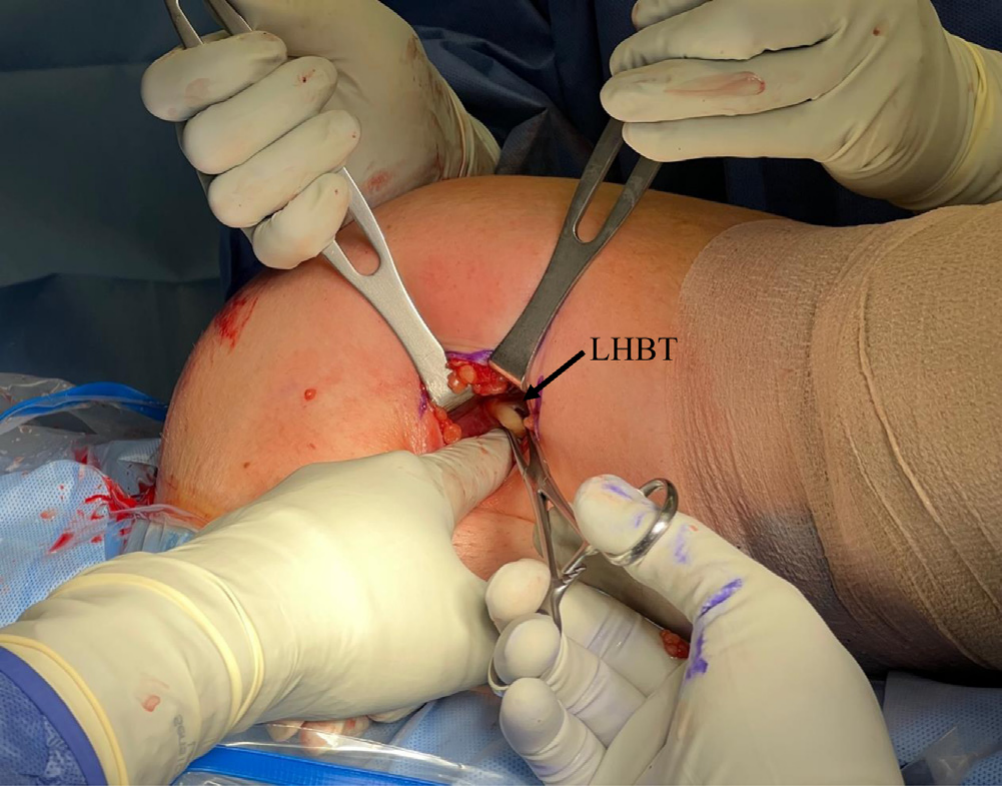
In a left shoulder in the lateral decubitus position, Army-Navy retractors are placed deep to the pectoralis major in a left shoulder, and the long head of the biceps tendon (LHBT) is retrieved using an Allis clamp.

**Fig 5:**
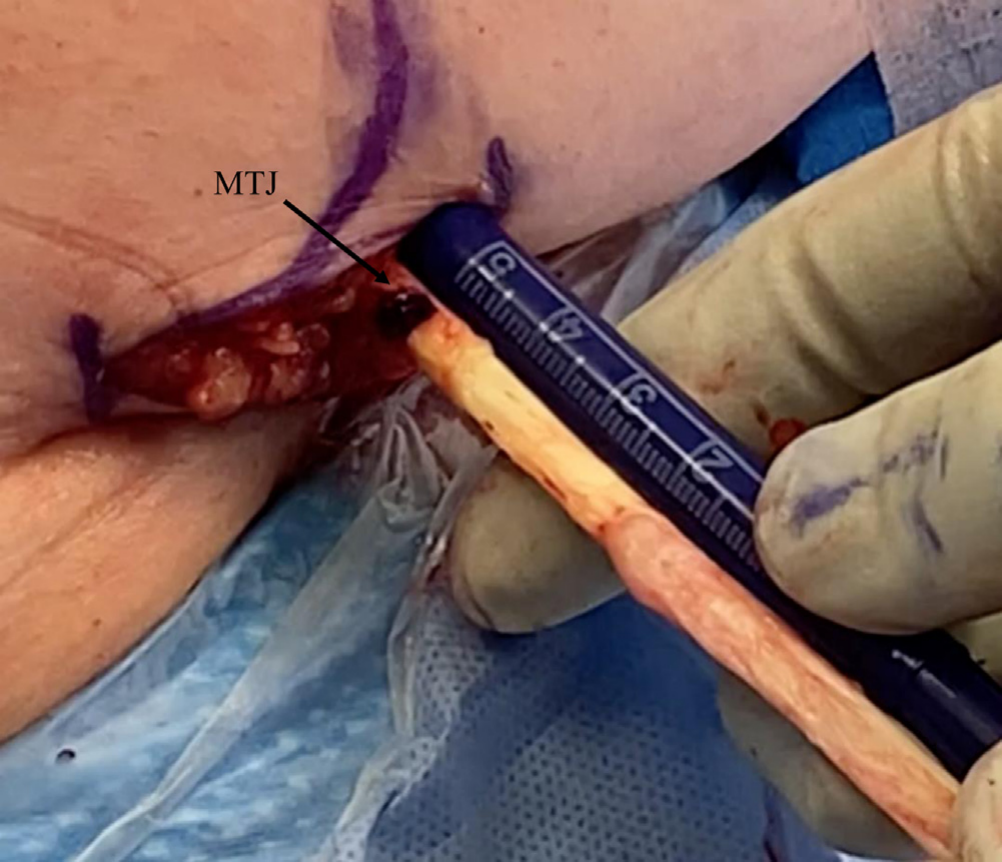
In a left shoulder in the lateral decubitus position, the inflamed synovial tissue overlying the long head of the biceps tendon is debrided sharply using tenotomy scissors. The tendon is then marked at the musculotendinous junction (MTJ), and an additional mark is made on the tendon 3 cm proximal to this location.

**Fig 6:**
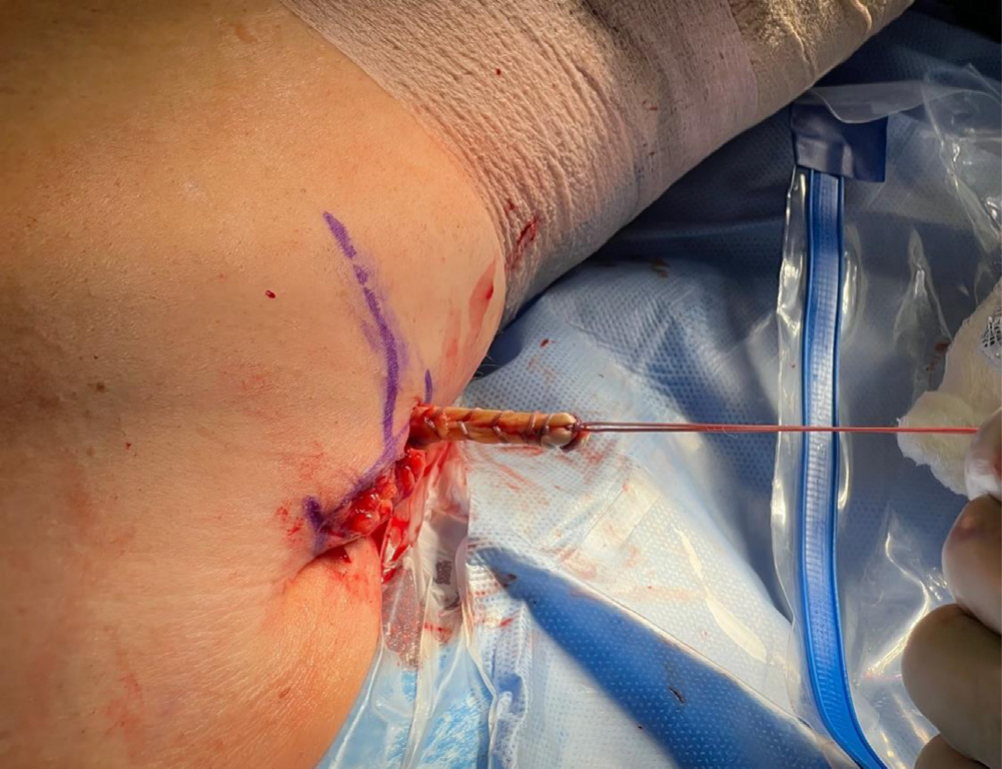
In a left shoulder in the lateral decubitus position, a FiberLoop is used to whipstitch the tendon over the 3-cm segment. The loop is then cut and the limbs are tied, making this a closed loop. A No. 15 blade scalpel is used to amputate the remaining biceps tendon proximal to the 3-cm mark.

The arm is extended 20° in an arm holder (Arthrex). The surgeon moves to the opposite side of the bed to complete the tenodesis. The incision is centered over the inferior border of the pectoralis major. A longitudinal 2.5-cm incision is first drawn with a skin marker, and a No. 10 blade scalpel is then used to incise the skin (Fig 2). Bovie electrocautery (Bovie Medical, Clearwater, FL) is used to achieve hemostasis and dissect through subcutaneous fat. Blunt finger dissection is then carried out down to the fascia overlying the pectoralis major. An Army-Navy retractor is placed, and the fascia in line with the inferior border of the pectoralis major is incised using Bovie electrocautery (Fig 3). Army-Navy retractors are then repositioned deep to the pectoralis major. The long head of the biceps tendon can be palpated within the bicipital groove. An Allis clap is used to retrieve the biceps tendon through the operative wound (Fig 4). Inflamed synovial tissue is debrided sharply using tenotomy scissors. The tendon is then marked at the musculotendinous junction using a marking pen, and an additional mark is made on the tendon 3 cm proximal to this location (Fig 5). A FiberLoop (Arthrex) is used to whipstitch the tendon over this 3-cm segment. The loop is then cut and the limbs are tied, making it a closed loop. Next, a No. 15 blade scalpel is used to amputate the remaining biceps tendon proximal to the 3-cm mark (Fig 6).

**Fig 7:**
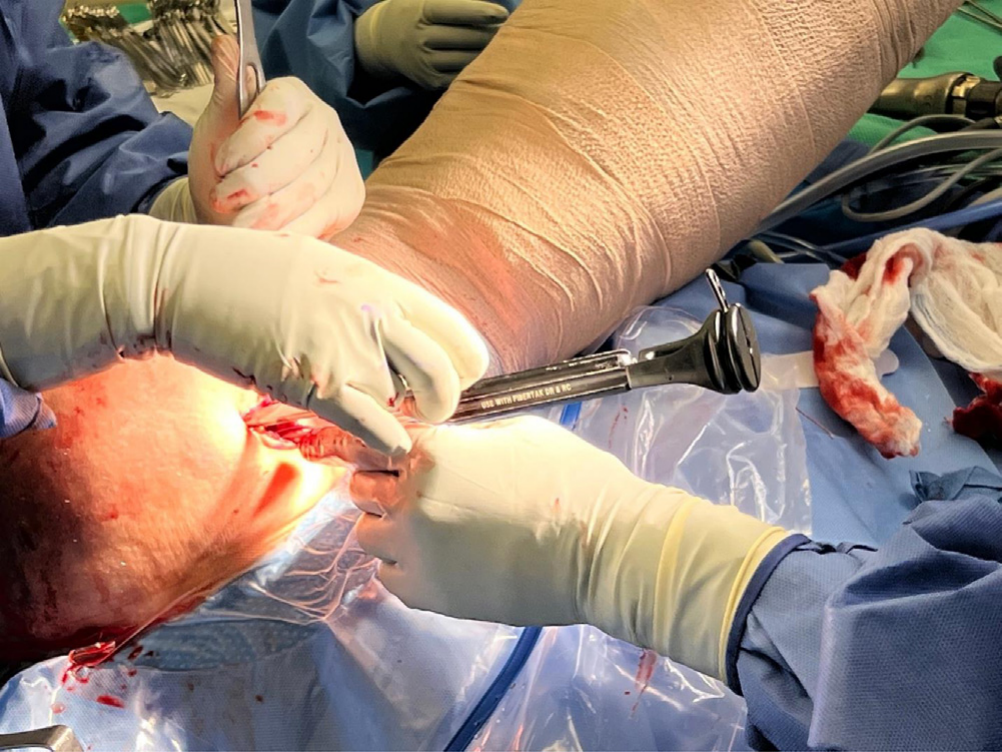
In a left shoulder in the lateral decubitus position, Bovie cautery has been used to create a small, 1-cm landing area on the anterior humerus for the drill guide. Through the guide, unicortical drilling of the tenodesis site is performed, and the 2.6-mm FiberTak is then tapped into place with a mallet on the anterior humerus.

**Fig 8:**
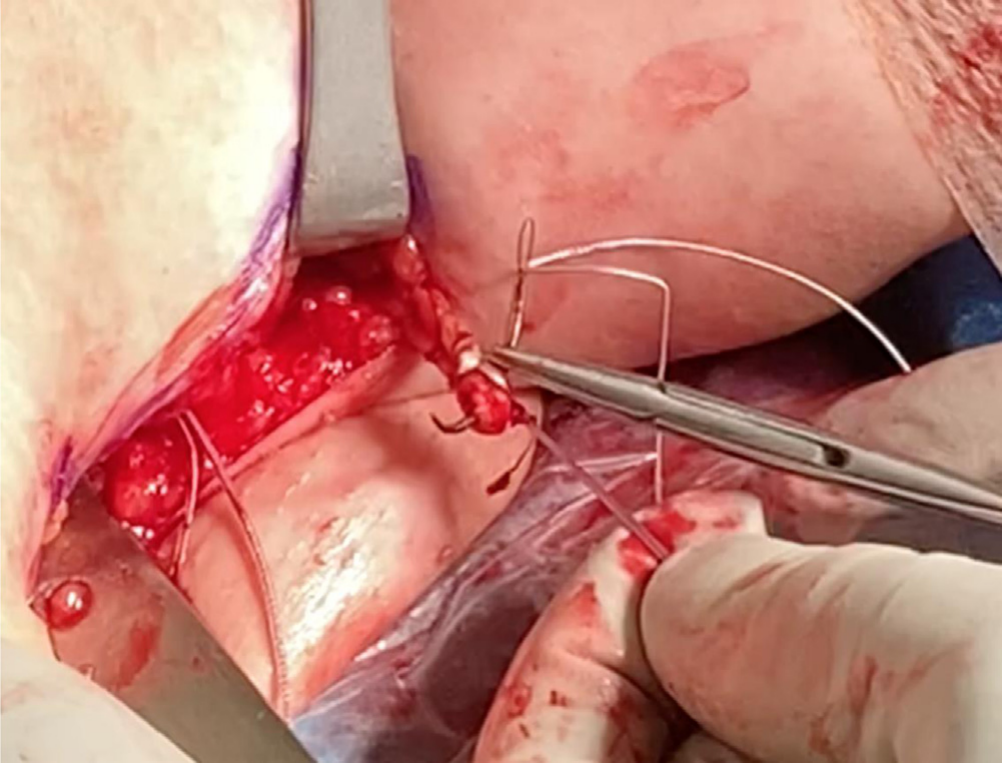
In a left shoulder in the lateral decubitus position, by use of a free needle, the repair suture from the anchor is passed through the last loop of the prepared biceps tendon in a left shoulder.

**Fig 9:**
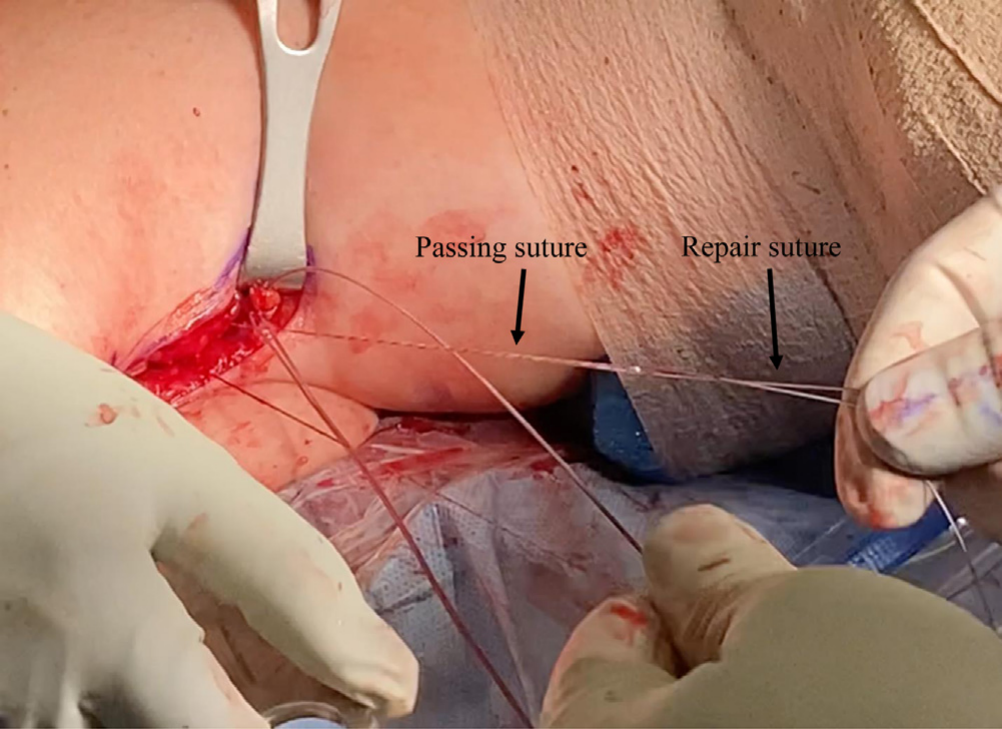
In a left shoulder in the lateral decubitus position, the repair suture is passed through the knotless anchor using the passing suture. This is tightened down, resulting in approximation of the biceps to the anterior humerus.

**Fig 10:**
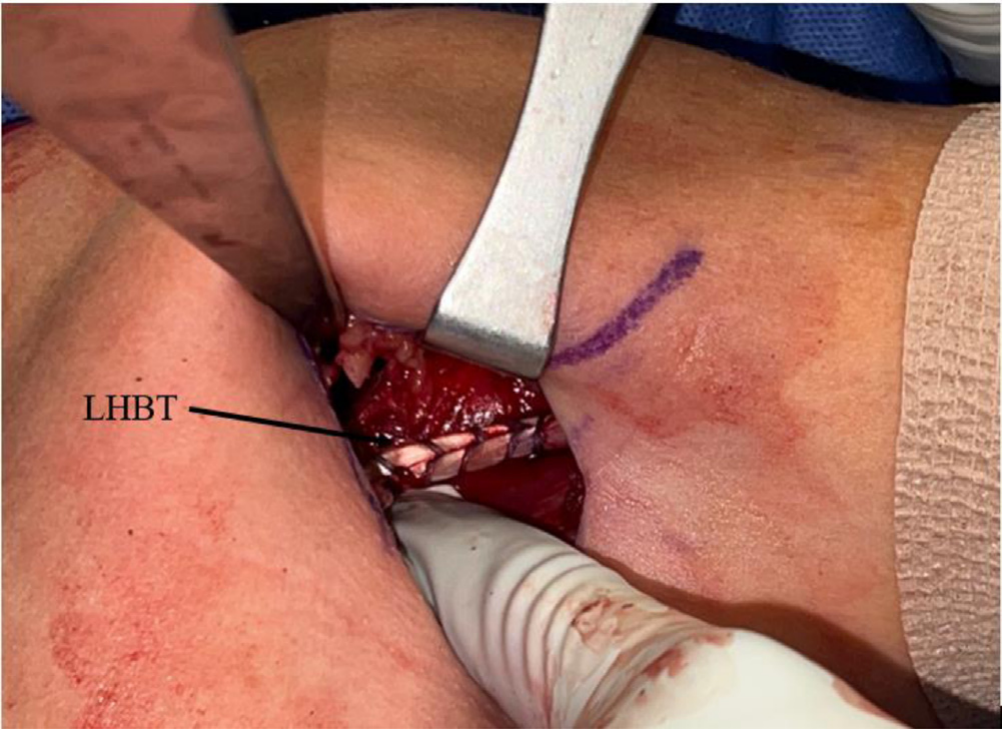
In the left shoulder in the lateral decubitus position, the long head of the biceps tendon (LHBT) has been secured to the anterior humeral tenodesis site with appropriate tension in the left shoulder.

**Fig 11:**
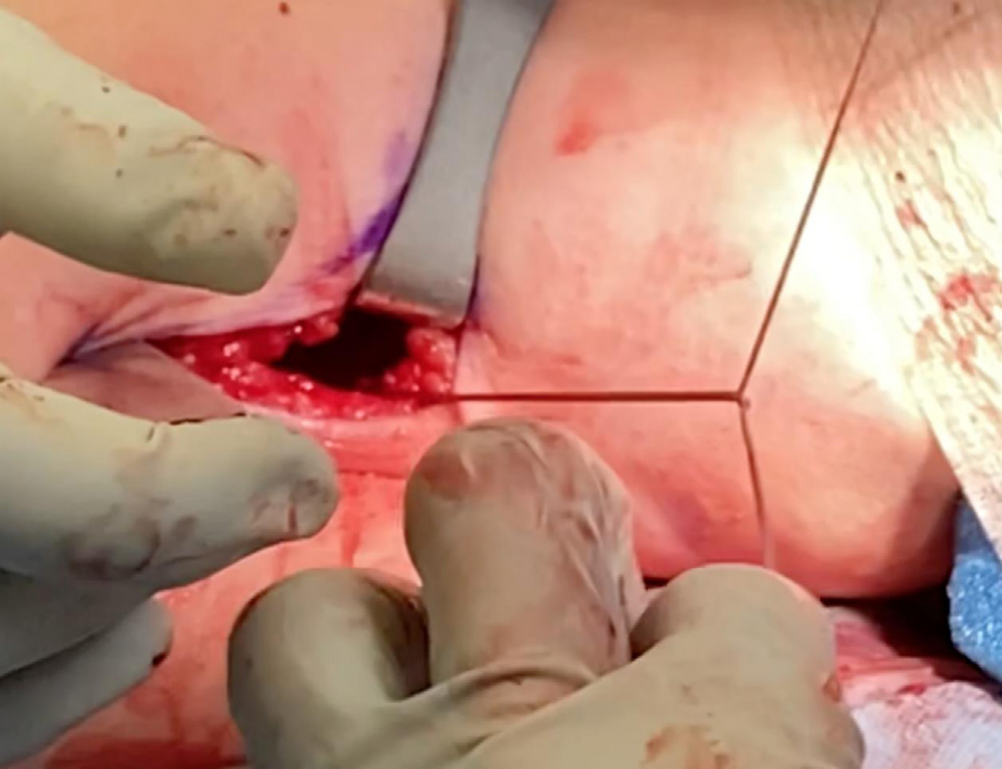
In the left shoulder in the lateral decubitus position, the anchor’s repair stitch and the FiberLoop ends are tied for backup fixation at the conclusion of the biceps tenodesis in the left shoulder.

We now turn our attention to the humerus. An Army-Navy retractor is placed deep to the pectoralis major, and a 90° bent Hohmann retractor is placed over the lateral aspect of the humerus just proximal to the pectoralis major insertion. Next, the pallium pectoralis is identified at the inferior portion of the bicipital groove. Bovie electrocautery is used to create a small, 1-cm “landing area” on the anterior humerus for the drill guide. The drill guide for the 2.6-mm Knotless FiberTak anchor (Arthrex) is then seated on the anterior humerus. A 2.6-mm drill is used to perform unicortical drilling, and the 2.6-mm Knotless FiberTak anchor is tapped into place with a mallet (Fig 7). The anchor is then set after removal of the drill guide. The repair suture from the anchor is passed through the last loop of the prepared biceps tendon using a free needle (Fig 8). The repair suture is then passed through the knotless anchor using the passing suture (Fig 9). The suture is tightened down, resulting in approximation of the biceps to the anterior humerus (Fig 10). The repair stitch and FiberLoop ends are then tied for backup fixation (Fig 11). Excess suture is cut. The wound is irrigated, and the skin is closed in a layered fashion (Video 1).

Postoperatively, the patient is required to wear a sling for 4 weeks. Physical therapy begins on postoperative day 1 or 2, focusing on passive range of motion initially, with eventual progression to active motion and strengthening. Full clearance is expected at 12 weeks postoperatively.

## Discussion

Although many different strategies for biceps tenodesis provide good patient outcomes and satisfactory results, we present a technique that has strengths in various areas. Using the subpectoral approach decreases the risk of persistent pain, recurrence, and revision seen with alternative locations while providing a consistent length-tension relation.^6^^,^^19^ The use of specific anatomic landmarks and measurements provides a consistent length-tension relation.^7^ The small, 2.6-mm all-suture anchor mitigates risks associated with other fixation devices without sacrificing strength of fixation. The knotless anchor provides efficiency and ease for the surgeon, as well as a smaller incision, resulting in better cosmesis for the patient (Table 1).

**Table 1:** Advantages and Disadvantages

Advantages
Elimination of bicipital groove pain
Lower reoperation rates than other similar techniques
Knotless anchor technique, eliminating risk of reliance on knot strength and pullout
Fast, easy, simple, reproducible technique
No need for step of tendon sizing
No need to find drill hole for tendon insertion
Disadvantages
Open approach with additional scar
Potential for neurovascular injury

Unlike tenotomy, biceps tenodesis provides the benefits of length-tension preservation, maintenance of elbow flexion and supination strength, and prevention of cosmetic deformity.^3^^,^^4^ Tenodesis techniques that localize the fixation above or within the bicipital groove and those that do not address the biceps sheath are associated with persistent pain thought to be due to inflamed tenosynovium, neural elements, or residual pathologic proximal tendon.^5^^,^^6^ Furthermore, tenodesis sites proximal to or within the bicipital groove are linked to higher surgical revision rates.^7^, ^8^, ^9^ Subpectoral tenodesis may provide better patient satisfaction. Although some proximal tenodesis techniques may be performed arthroscopically, open subpectoral biceps tenodesis has been found to have an overall low incidence of complications.^5^ Furthermore, the incision size may be minimized by using smaller fixation devices and reproducible techniques to identify the appropriate location. Authors have created guidelines for reproducing the proper length-tendon relation of the biceps when using this approach.^20^^,^^21^ The senior author (N.M.) uses the landmark described by Moore et al.,^8^ the pallium pectoralis, to identify the appropriate location for tenodesis in relation to the number of tendon sutures from the myotendinous junction. This fascial sleeve connects the top of the pectoralis major to the medial border of the humerus, about 2 cm distal to the transverse humeral ligament at the metadiaphyseal junction of the humerus. This location not only provides the benefits of subpectoral fixation, as well as a reproducible landmark, but also avoids the risk of fracture seen with more distal diaphyseal fixation.^15^

An important aspect of this technique is the use of a small all-suture knotless anchor for onlay fixation of the biceps tendon. Fixation strategies align within 2 broad categories of inlay and onlay tenodesis. Interference screws are typically used for inlay techniques, with a variety of anchors or buttons being used for most onlay techniques. Concern has recently grown regarding the large drill hole creating a stress riser, predisposing to humeral fracture.^14^, ^15^, ^16^ Drill hole size has been found to directly correlate to the risk of fracture.^14^^,^^15^ Additionally, other complications including implant failure and screw reactions have been reported.^10^^,^^14^ Using a smaller hole for fixation along with an all-suture anchor helps to mitigate these risks. Tendon diameter sizing is also needed for inlay techniques, making onlay techniques more efficient in this respect as well. Our technique uses a 2.6-mm drill hole for anchor insertion, much smaller than that used for typical interference screws and even other onlay button devices.

Initially, there was some concern that strength of fixation and patient outcomes may be sacrificed with the use of an onlay technique. Recent literature has supported onlay fixation to have comparable strength to inlay fixation.^18^ Multiple studies comparing the 2 techniques have found no significant differences in postoperative outcomes and patient satisfaction.^5^^,^^6^^,^^20^

The all-suture nature of the aforementioned implant mitigates the risk of patient reactions, as documented for some other anchor types.^5^^,^^12^ Metal button implants may also possess this benefit. When using most anchors, the surgeon must not only create a larger hole but then find the same hole for implant insertion, as is the case with many screw fixation techniques as well. This can prove difficult when paired with bleeding, inadequate visualization and/or retraction, or large body habitus. When using the anchor described in our technique, the surgeon drills and inserts the anchor through the provided guide, eliminating this potential difficulty.

It is important to note that strength of fixation is not sacrificed with the use of the all-suture knotless anchor. The knotless FiberTak has been shown to have no statistically significant difference in biomechanical strength in comparison to knotted anchors in labral repair.^8^ Lacheta et al.^14^ found no difference in load to failure when comparing a metal button with a 1.8-mm all-suture FiberTak anchor in subpectoral biceps tenodesis. It is interesting to note that the mode of failure was tendon tearing in 56% of cases and knot failure in 44% of cases in the suture anchor group, in comparison to tendon tearing in 100% of cases in the button group. There is known variability in knot strength even in repairs performed by experienced surgeons.^22^ In addition, unlike other all-suture anchors, the knotless mechanism voids the time required for tying of knots, as well as the risk of knot slippage and potential tendon-anchor interface gapping. This provides additional efficiency and consistency with the technique.

Our technique provides the benefits of a subpectoral tenodesis site, an onlay technique, and consistent anatomic landmarks. The small, 2.6-mm all-suture anchor mitigates risks and difficulties associated with other fixation devices without sacrificing strength of fixation. The ease and efficiency of the knotless all-suture anchor are apparent.

## Supplementary Data

ICMJE author disclosure forms

Video 1 Subpectoral biceps tenodesis using an all-suture knotless anchor technique in a left shoulder.
